# Berberine ameliorates nonalcoholic fatty liver disease by a global modulation of hepatic mRNA and lncRNA expression profiles

**DOI:** 10.1186/s12967-015-0383-6

**Published:** 2015-01-27

**Authors:** Xinlu Yuan, Jie Wang, Xiaoyan Tang, Yixue Li, Pu Xia, Xin Gao

**Affiliations:** Department of Endocrinology and Metabolism, Zhongshan Hospital, Fudan University, Shanghai, China; Key Laboratory of Systems Biology, Shanghai Institutes for Biological Sciences, University of Chinese Academy of Sciences, Shanghai, China

**Keywords:** Berberine, NAFLD, lncRNAs, *MRAK052686*, *Nrf2*

## Abstract

**Background:**

Nonalcoholic fatty liver disease (NAFLD) is a common liver disorder that currently lacks effective treatment. Berberine (BBR), a botanic compound isolated from traditional Chinese medicine, exhibits a potent therapeutic potential for the metabolic disease. The current study aimed to understand the mechanisms underlying the therapeutic effect of BBR in NAFLD.

**Methods:**

We performed systematical analyses on hepatic expression profiles of mRNAs and long noncoding RNAs (lncRNAs) in a high-fat diet (HFD)-induced steatotic animal model with or without BBR treatment. The study was conducted by using the methods of bioinformatics, including hierarchical clustering, gene enrichment and gene co-expression networks analysis. The effect of BBR on the expression profile of some interesting genes was confirmed by quantitative RT-PCR and further studied in a human hepatic cell line, Huh7.

**Results:**

We found that a large group of genes including 881 mRNAs and 538 lncRNAs whose expression in the steatotic liver was reversed by BBR treatment, suggesting a global effect of BBR in modulating hepatic gene expression profiles. Among the BBR-regulated genes, we identified several modules and numerous significant genes that were associated with liver metabolism and NAFLD-related functions. Specifically, a conserved lncRNA, *MRAK052686*, was found strongly correlated with the antioxidant factor *Nrf2*, and both genes were down-regulated by the steatotic liver. Moreover, the reduced expression of *MRAK052686* and *Nrf2* was completely reversed by BBR treatment, suggesting a new mechanism accounting for the therapeutic effect of BBR.

**Conclusions:**

The findings for the first time provide a new genetic insight into the pharmaceutical mechanism of BBR in protecting against NAFLD.

**Electronic supplementary material:**

The online version of this article (doi:10.1186/s12967-015-0383-6) contains supplementary material, which is available to authorized users.

## Background

Nonalcoholic fatty liver disease (NAFLD) has become a worldwide health problem that influences approximately 20–30% of the general population and its prevalence is continuously increasing [1,2]. NAFLD is closely associated with obesity, hyperlipemia and type 2 diabetes, and thus often regarded as atypical hepatic manifestation of the metabolic syndrome [3]. Under certain circumstances, NAFLD can progress from hepatic steatosis to steatohepatitis, leading to an increased susceptibility of cirrhosis and hepatocellular carcinoma [2]. However, there is still a limitation in both mechanistic understanding and clinical management of the disease. Besides life style intervention, several medications, e.g., Metformin, vitamin E and Pioglitazone, are currently applied in the clinic for treatment of NAFLD [4,5]. However, these medications often exhibited a limited efficacy and considerable side effects. Therefore, much effort has been focused on the development of new therapeutics for NAFLD.

Berberine (BBR), isolated from the herb *Rhizoma Coptidis*, has been widely used in traditional Chinese medicine to treat diarrhea and many other inflammatory diseases [6]. Recent studies have revealed a novel therapeutic role of BBR in metabolic disorders, including obesity and diabetes [7,8]. BBR can function as a cholesterol-lowering drug via a unique mechanism distinct from statins [9]. These findings suggest a potential therapeutic activity of BBR for NAFLD. Indeed, both *in vitro* and *in vivo* studies from our previous work and many others have shown that BBR profoundly inhibited lipid synthesis and accumulation in hepatocytes, attenuated hepatic steatosis and hyperlipidemia, and prevented the progression of NAFLD [10-14]. Mechanistically, the therapeutic activity of BBR has been suggested to attribute to its effects of overcoming insulin resistance, reducing endoplasmic reticulum (ER) stress and regulating the signaling pathways, such as the AMPK and JNK pathways [15-18]. More recently, BBR has been shown to modulate gut microbiota, which also account for the therapeutic effect of BBR on the metabolic diseases [19]. It thus appears that multiple mechanisms are involved in the therapeutic effect of BBR, leading us to hypothesize that BBR may have a global effect in modulating gene expression profile in the liver and thereby protecting against hepatic steastosis.

Long noncoding RNAs (lncRNAs) are a novel class of RNA transcripts that are more than 200 bp in length and have little or no protein-coding capacity [20]. According to chromosomal position relative to coding RNAs, lncRNAs are mainly grouped into intergenic, intronic, sense and antisense non-coding RNAs [21]. Most lncRNAs show moderately evolutional conservation and specific transcription [22]. Recently, lncRNAs have been shown widely involved in epigenetic regulation via their direct or indirect interactions with chromatins [23]. Acting as important regulatory molecules, miscellaneous lncRNAs are critically complicated diverse biological processes from normal development and human diseases [24,25]. However, there are little studies on lncRNA in NAFLD. In an attempt to further understand the mechanisms underlying the therapeutic effect of BBR, we performed systematical analyses on hepatic expression profiles of mRNAs and lncRNAs in a high-fat diet (HFD)-induced steatotic animal model with or without BBR treatment as reported in the present study.

## Materials and methods

### Animals

Animal studies were approved by the Animal Use and Care Committee of Fudan University and were in conformity with the US Public Health Service Policy on Humane Care and Use of Laboratory Animals. Total 24 healthy male Sprague-Dawley rats weighing around 200 g were obtained from the Animal Development Center, Chinese Academy of Sciences, Shanghai. All rats were acclimated for 1 week before initiation of the experiment and maintained on a 12/12 h light/dark cycle with free access to food and water. The animals were divided to the following three groups (8 rats per group): (i) Control group (ND group), received a regular rodent chow diet (62.3% carbohydrate, 12.5% fat and 24.3% protein in total calories); (ii)HFD group, fed a HFD (32.6% carbohydrate, 51.0% fat and 16.4% protein) for 24 weeks; and (iii) HFD + BBR group, after 8 weeks of HFD feeding, rats were orally treated with BBR (Sigma, St. Louis, USA) at a dose of 200 mg/kg/day via a lavage needle and fed on HFD feeding for 16 weeks. Animals received intraperitoneal glucose tolerance tests (IPGTT), weekly measurements of body weight and food intake, and monthly tests of fasting serum insulin and glucose as described previously [12]. Hepatic lipids were extracted according to the method of Folch et al. [26] and triglyceride content was tested as described previously [27]. Fasting serum levels of total cholesterol and low-density lipoprotein cholesterol (LDL-c) were measured using an Amplex Red Cholesterol Assay Kit (A12216, Molecular Probes Invitrogen Detection Technologies) according to the manufacturer’s protocol.

### Histological examination

All liver samples were collected after animals were sacrificed in 24th week of the treatment and subsequently fixed in phosphate-buffered 10% formalin. The right lateral lobule of each liver was embedded in paraffin blocks and stained with hematoxylin and eosin (H&E) examining the pathologic features of the liver.

### Microarrays of mRNAs and lncRNAs

Arraystar Rat LncRNA microarrays (accession in NCBI Gene Expression Omnibus, http://www.ncbi.nlm.nih.gov/geo: GPL15690) were used for measuring the expression profiles of lncRNAs and mRNAs in total 15 rats (i.e., 3 groups at 5 replicates). The sample preparations and microarray hybridization were performed based on the manufacturer’s protocols. Briefly, 1 μg of total RNA from each sample was amplified and transcribed into fluorescent cRNA using Agilent’s Quick Amp Labeling kits according to the manufacturer’s protocol (version 5.7, Agilent Technologies). The labeled cRNAs were hybridized onto the Rat LncRNA Arrays (4×44K, Arraystar, Rockville, USA). After washing, the arrays were scanned by the Agilent Scanner G2505B. Raw signal intensities were normalized in quantile method by GeneSpring GX v11.5.1. After filtration of low intensity RNAs, those RNAs with flags in Present or Marginal at least 1 out of 15 samples were chosen for further analysis. Differentially expressed genes were identified by using the Limma package [28]. The threshold set for significantly differential genes was an absolute log value of fold change ≥ 0.6 and a p value < 0.05.

### Hierarchical clustering and gene enrichment analysis

We took Euclidean distance with a complete linkage to hierarchically cluster the genes across the samples. Unsupervised hierarchical clustering and the visualization of heatmap were performed using the R platform (http://www.r-project.org/). Gene function enrichment analysis was performed by using DAVID [29]. The significance of enrichment was calculated by hypergeometric test and adjusted by Benjamini method. GO (gene ontology) and KEGG (kyoto encyclopedia of genes and genomes) were accessed from the databases of http://www.geneontology.org/ and http://www.genome.jp/, respectively [30,31].

### Gene module-trait relationships

We analyzed associations between gene modules and the traits by using WGCNA (weighted correlation network analysis) package [32]. The package assessed module-trait relationships by correlating of each module eigengene (the first principal component of gene expression in the module) with the traits.

### Construction of gene co-expression networks

We constructed and analyzed the co-expression network of lncRNAs and mRNAs using WGCNA package. The weight of edges was scaled by a soft power of correlation coefficient of parewise genes, and the soft power was set to 16 after stimulation. The visualization and layout of network were achieved by cytoscape software [33].

### Prediction for protein-coding potential and interaction propensity of lncRNAs

The protein-coding potential of lncRNAs was predicted by PhyloCSF which calculates codon substitution frequencies (CSF) determining whether a multispecies nucleotide sequence is conserved protein-coding region [34]. The more positive value of CSF indicates a higher potential to code protein, vice versa. Multiple alignments of 8 vertebrate genomes are downloaded from UCSC (http://genome.ucsc.edu/). We used catRAPID to predict the interaction propensity of lncRNAs with proteins [35]. The software catRAPID computes interaction propensity (IP, a measure based on physico-chemical profiles of ribonucleoprotein complexes) and discriminative power (DP) for assessing the potential of interaction. DP above 75% represents highly confident IP.

### Identification of human homologous of the lncRNA *MRAK052686*

According to genome-wide alignment from UCSC (http://genome.ucsc.edu) and chromosomal position, we found that human *MRAK052686* is located around *ZBTB20*. A direct searching of *MRAK052686* in human genome indicated the same chromosomal location as mentioned above. By searching this human locus in ENSMBL database (http://www.ensembl.org) and then aligning sequences, we found 6 of 11 non-coding transcripts with a high similarity to rat *MRAK052686* (Additional file 1: Table S1 and Additional file 2: Figure S1). These six lncRNAs were thus regarded as human homologues of *MRAK052686*.

### Cell culture, treatment and intracellular lipid staining

Huh7, a human hepatoma cell line, was obtained from American Type Culture Collection (Manassas, VA) and routinely cultured in DMEM supplemented with 10% fetal bovine serum. For treatment, a stock solution of 100 mM oleic acid with 11% (w/v) fatty acid-free BSA was prepared. When reaching a 70% confluence, cells were washed with PBS and exposed to 100 μM OA for 24 h in the presence or absence of 10 μM BBR. For intracellular lipid staining, cells were washed with PBS three times and fixed with 4% paraformaldehide for 30 min. Intracellular lipids were stained with Nile Red (Sigma, St. Louis, USA) for 15 min and nuclei stained with DAPI for an additional 5 min. Images were photographed with an inverted fluorescence microscope (Leica DM2000, Wetzlar, Germany).

### Real-time quantitative PCR

All rats were euthanized and their livers were collected after 24 weeks treatment. Total RNA was isolated from liver tissues using Trizol reagent (Invitrogen, Carlsbad, CA). cDNA was synthesized by reverse transcription in a 20 μL reaction system using PrimeScript kit (Takara, Tokyo, Japan). The reverse transcription reactions were conducted at 37°C for 15 min and inactivated at 85°C for 30s using an automatic thermocycler according to manufacturer’s instruction. Real-time quantitative PCR (RT-qPCR) was performed with an Applied Biosystems 7500 using SYBR Premix EX Taq (Takara, Tokyo, Japan). A total 10 μL reaction mixture contained of 5 ng sample cDNA, 5 μM specific forward and reverse primers, and 5 μL SYBR Premix EX Taq. The sequences of primers used for RT-qPCR are shown in Additional file 1: Table S2. The PCR reaction was consisted of an initial denaturation cycle at 95°C for 10 min and 40 amplification cycles of 95°C for 15 s and 60°C for 1 min. The relative gene expression was calculated using the 2^-ΔΔCt^ method [27]. The same reaction was carried out in triplicate with β-actin as an internal control. Experiments of each condition were conducted in 3 replicates.

### Statistical analysis

All data were presented as mean ± SEM. Significance was assessed by one-way ANOVA followed by Tukey’s test and LSD for multiple comparisons. Chi-square test was used to assess the significance of variables in the contingency table. All statistics were performed in R platform. A value of p < 0.05 was considered statistically significant.

## Results

### BBR ameliorates hepatic steatosis in HFD-fed rats

**Figure 1:**
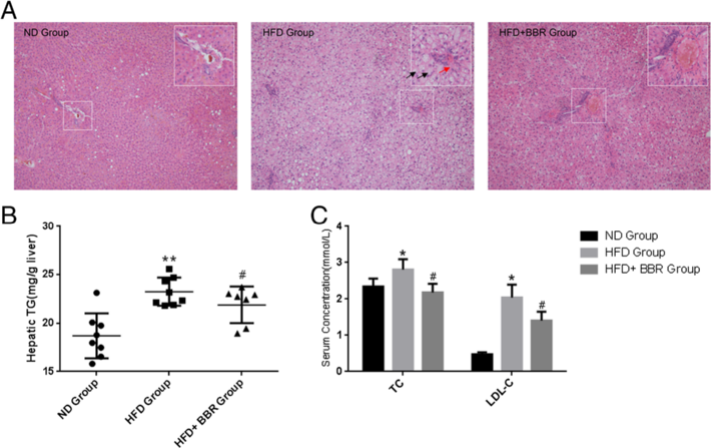
**BBR ameliorates hepatic steatosis in HFD-fed rats. (A)**The livers isolated from rats fed with a normal diet (ND), high-fat diet (HFD) or HFD plus Berberine (HFD + BBR)were stained with hematoxylin and eosin. Photographs are at 200 × magnification. A large magnification is shown in the white box. Ballooning cells and Mallory bodiesare shown in black and red arrows, respectively. **(B)** Hepatic TG contents, and **(C)** serum TC and LDL-c levels were measured in the experimental animals as described in “Methods”. Data are mean ± SEM. * p < 0.05, ** p < 0.01 versus ND; # p < 0.05 versus HFD (assessed by one-way ANOVA followed by Tukey’s multiple comparison tests). BBR, berberine; HFD, high-fat diet; ND, normal diet; TG, triglyceride; TC, cholesterol.

BBR has been reported to prevent the development of NAFLD in animal models [12,36,37]. To confirm the therapeutic effect of BBR, we examined the effect of BBR on the liver in HFD-induced obese rats. As shown in Figure 1, HFD-fed animals exhibited moderate to severe hepatic steatosis with typical histological features of cell swelling and large amount of fat accumulations within hepatocytes. Remarkably, HFD-induced steatosis was evidently ameliorated by BBR treatment, as reflected in a significant reduction of ballooning cells and Mallory bodies (Figure 1A). Moreover, BBR treatment significantly lowered hepatic TG contents and serum levels of TC and LDL-c by 13.7%, 28.1% and 41.9%, respectively, in comparison with the HFD-fed animals (all p < 0.05, Figures 1B and 1C).

### BBR systematically modulates hepatic gene expression

**Figure 2:**
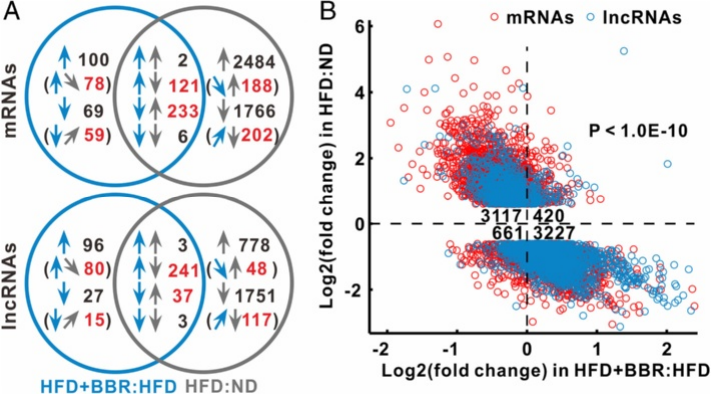
**BBR systematically regulates hepatic gene expression. (A)** A Venn diagram of differentially expressed genes in the experimental rats. The cyan and grey arrows represent the comparisons of HFD + BBR with HFD group (HFD + BBR:HFD) and HFD with ND group (HFD:ND), respectively. The upward arrows indicate up-regulation and downward, down-regulation. Sloped arrows in brackets indicate genes whose expression is not significantly altered but meets our criteria of BBR-regulated genes. Numbers in brackets indicate a subset of the number immediately above. The red number is the count of BBR-regulated genes. **(B)** BBR reversed most differentially expressed genes in HFD relative to ND group. The numbers in the center indicate the count of genes in corresponding quadrant. The p value was calculated with the Chi-squared test.

In an attempt to understand the molecular basis of the efficacy of BBR, we performed a microarray-based analysis of hepatic gene expression profiles in ND, HFD and HFD + BBR treated animals. After normalization and filtration of microarray data, total 14, 319 mRNAs and 9, 531 lncRNAs were identified for further analysis. By comparison of HFD + BBR with HFD group, we identified 531 and 407 differentially expressed mRNAs and lncRNAs, respectively (Figure 2A). In addition, the second comparison of HFD with ND group revealed that 4, 612 mRNAs (including 2, 719 up-regulated and 1, 893 down-regulated mRNAs) and 2, 813 lncRNAs (consisting of 818 up-regulated and 1, 995 down-regulated lncRNAs) were differentially expressed, of which 362 mRNAs and 284 lncRNAs were overlapped with the first comparison (Figure 2A). Among the overlapped and reversely expressed genes in two comparisons, BBR significantly up-regulated more lncRNAs (241 lncRNAs vs. 121 mRNAs) and down-regulated more mRNAs (233 mRNAs vs. 37 lncRNAs) (Figure 2A, p < 1.0E-10, chi-squared test). Moreover, among the 7,425 genes whose expression was altered in HFD group, 6,344 (85.4%) genes including 2,399 lncRNAs were reversed towards a normal expression profile after BBR treatment (Figure 2B, p < 1.0E-10). In addition, by comparing HFD and HFD + BBR with ND group, respectively, we found much fewer differences in mRNA and lncRNA expression between HFD + BBR and ND than that between HFD and ND groups (476 mRNAs and 374 lncRNAs versus 2131 mRNAs and 1402 lncRNAs, Additional file 3: Figure S2). Taken together, the data suggest a potent effect of BBR on a global regulation of hepatic gene expression in the steatotic liver trending towards a normal profile, including abundant lncRNAs.

### Expression profiles of BBR-regulated genes

In order to further characterize the effect of BBR on hepatic gene regulation, we specifically grouped the genes whose expression was potentially regulated by BBR. To this end, we used the following criteria to determine the genes as BBR-regulated genes: (i) the gene expression was inversely changed in two comparisons, and (ii) absolute log value of fold change ≥ 0.6 in the first comparison between HFD + BBR and HFD groups. Accordingly, total 881 mRNAs and 538 lncRNAs were identified as BBR-regulated genes whose expression patterns were reversed after BBR treatment in comparison to HFD group (Figure 2A, Additional file 4: Table S3).

**Figure 3:**
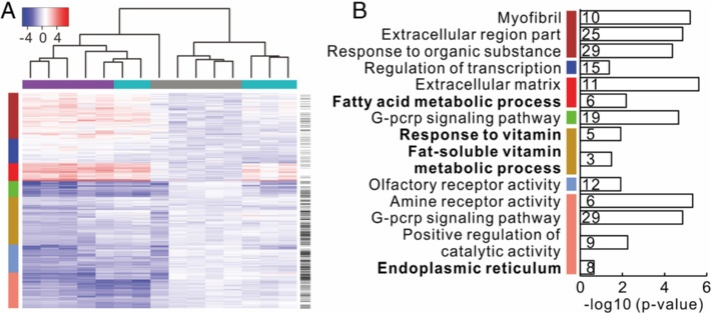
**Hierarchical clustering and functions enrichment analyses of BBR-regulated genes. (A)** Each column represents samples from ND (grey), HFD (purple) or HFD + BBR (cyan) group. Each row indicates genes, in which lncRNAs are labeled with black bars on the most right column. Each cell of the heat-map reflects expression value above or below the pool of control rats fed ND. The legend of expression value is shown on the upper left. Based on gene clustering tree (Additional file 5: Figure S3), BBR-regulated genes are divided into seven modules, indicating with the most left color column: brown, blue, red, green, dark golden, light blue and salmon. **(B)** Representative enriched functions for seven BBR-regulated modules. The number on the right of vertical axis indicates the count of enriched genes in corresponding function. The NAFLD-related functions highlight in bold. The color bars on the left of vertical axis represent the modules. More details of the enriched functions for each module are shown in Additional file 5: Table S3.

For assessing the association of lncRNAs with mRNAs, we performed hierarchical clustering of BBR-regulated genes across the three experimental groups. HFD + BBR group exhibited a similar expression profile of BBR-regulated genes to the ND group (Figure 3A). Furthermore, we divided BBR-regulated genes into seven modules (termed as BBR-regulated modules) based on gene clustering trees (Additional file 5: Figure S3). These modules represent distinct expression patterns. In these modules, lncRNAs were not uniformly distributed, whereas lncRNAs were evidently enriched in the dark golden and light blue modules (Figure 3A).

Based on the above seven BBR-regulated modules, we assessed the correlation of NAFLD-related traits with the module eigengenes using WGCNA package [32]. We found that salmon module was mostly correlated with triglyceride metabolism and red module associated with lipoproteins (Additional file 6: Figure S4). Of note, function enrichment analysis by using DAVID [29] indicated that the salmon and red modules are likely associated with NAFLD, including the genes involved in endoplasmic reticulum (ER) function and fatty acid metabolism (Figure 3B, and Additional file 1: Table S4). In addition, mRNAs in dark golden module is also related to NAFLD, including the genes involved in fat-soluble vitamin metabolic process. Collectively, these data indicate that a large number of BBR-regulated genes including lncRNAs are indeed functionally associated with NAFLD (especially those from salmon, red and dark golden modules).

### The lncRNA *MRAK052686* is a significant BBR-regulated gene

**Figure 4:**
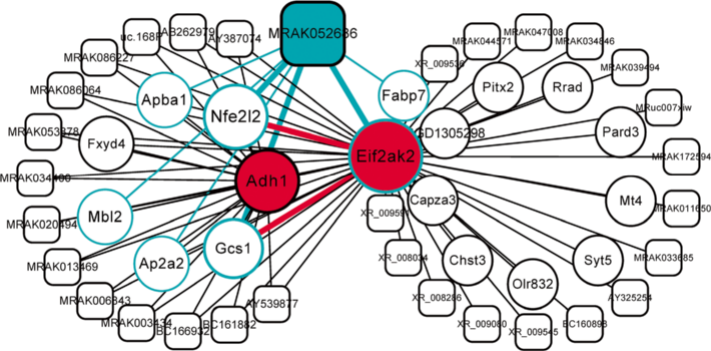
**Co-expression sub-networks derived from enriched genes in dark golden module.** Nodes of sub-networks consist of enriched genes and their first neighboring genes. The threshold for edges of co-expression network is 0.25 for weighted correlation. The red genes, *Eif2ak2* and *Adh1,* were identified by enrichment analysis showing in Figure 3B. The potentially important lncRNA *MRAK052686* and its associated mRNAs are highlighted in cyan color. Rectangles and circles represent lncRNAs and mRNAs, respectively.

Given the numerous BBR-regulated genes as identified above, we assessed gene significance by analyzing association of gene expression with traits. The genes with a high significance for trait and strong correlation in module were characterized as potentially important genes. Accordingly, we identified two significant lncRNAs, *MRAK052212* and *MRAK080926*, from the salmon module that was highly correlated with triglyceride metabolism (Additional file 7: Figure S5A). The function annotation of associated mRNAs indicated that *MRAK052212* and *MRAK080926* may participate in lipid metabolism and ER to Golgi vesicle-mediated transport process (Additional file 1: Table S5). Furthermore, we identified another two most significant protein-coding genes from the red module, including *Lyz2* and *Tnfrsf18*, which were highly correlated with LDL trait (Additional file 7: Figure S5B). *Lyz2* is suggested to be associated with catabolic process in ER according to gene ontology (GO). Then, we analyzed co-expression sub-networks from the significant genes and their first neighbor genes in each NAFLD-related module. In the sub-networks derived from salmon and red modules, we identified three potentially important lncRNAs, including *AF034247*, *MRuc007jok* and *BC166462*, which were all connected with enriched protein-coding genes (Additional file 8: Figure S6). In the dark golden module, a sub-network was delineated based on two enriched and strong co-expressed genes, *Eif2ak2* (also known as *Prkr*) and *Adh1* (Pearson’s correlation coefficient, R = 0.59, p = 0.02). Interestingly, in this sub-network we found an lncRNA, *MRAK052686* that was strongly associated with *Eif2ak2* (R = 0.59, p = 0.022) and *Nrf2* (also known as *Nfe2l2*, R = 0. 91, p = 2.7e-06) (Figure 4). Both *Eif2ak2* and *Nrf2* have been previously documented being associated with the pathogenesis and progression of NAFLD [38]. In addition, according to the KEGG Pathway Database, *Nrf2* was a direct downstream target of *Eif2ak2* in ER stress response pathways. Furthermore, the lncRNA *MRAK052686* was co-expressed with several other NAFLD-related genes, including the fatty acid binding protein *Fabp7* and *Gcs1,* which are involved in ER protein processing (Additional file 1: Table S5). Together, these results suggest that the lncRNA *MRAK052686* may play important roles in NAFLD by affecting the genes that regulate cellular stress response and protein processing in ER. Besides *MRAK052686*, we found more lncRNAs (accounting for 65%) connected to the NAFLD-related genes in sub-networks of dark golden and salmon modules (Figure 4 and Additional file 8: Figure S6), further indicating an important role of lncRNAs in NAFLD.

*MRAK052686 is* located in 3’UTR of protein-coding gene *Zbtb20*, which is an important regulator of glucose homeostasis and has been reported to associate with liver dysfunction [39]. However, we found no difference in *Zbtb20* expression between HFD + BBR and HFD groups (data not shown) and no correlation of *Zbtb20* to *MRAK052686*. This suggests that *MRAK052686* is a transcript independent of *Zbtb20*. Further analysis indicated that codon substitution frequency of *MRAK052686* is strongly negative (Additional file 9: Figure S7). Moreover, we found no protein sequence significantly matched with *MRAK052686* from NCBI reference protein database (data not shown), further suggesting that *MRAK052686* has no or little potential to code protein.

### BBR restores the decreased expression of *MRAK052686* and *Nrf2* in steatotic liver

**Figure 5:**
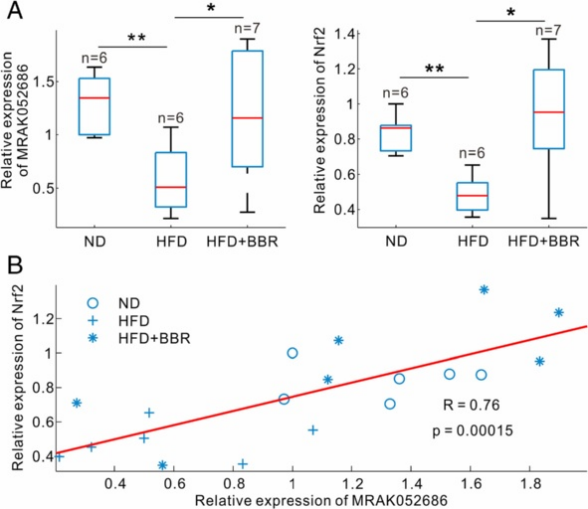
**Co-expression of** ***MRAK052686*** **and** ***Nrf2*** **across three groups of the rats. (A)** Levels of *MRAK052686* and *Nrf2* expression in livers were measured by RT-qPCR and normalized by β-actin expression. Significance was assessed by one-way ANOVA followed by Tukey’s multiple comparison tests. Data are shown as mean ± SEM (n ≥ 4). Pearson’s correlation coefficient (R) and corresponding p value of the fitted line are shown in **(B)**.

Having identified the lncRNA *MRAK052686* and the related gene *Nrf2* from the NAFLD-related modules, we wanted to clarify whether their expression was correlated in NAFLD and whether regulated by BBR treatment. By conducting RT-qPCR assays, we confirmed that both *MRAK052686* and *Nrf2* expression was significantly down-regulated in the livers of HFD group compared with the controls (p < 0.01, Figure 5A). Moreover, the reduced expression of *MRAK052686* and *Nrf2* was markedly reversed by BBR treatment (p < 0.05, Figure 5A). In addition, a significant positive correlation between hepatic expression levels of *MRAK052686* and *Nrf2* existed across ND, HFD and HFD + BBR groups (R = 0.76, p = 0.00015, Figure 5B), further supporting a strong co-expression profile of these two genes in the liver.

**Figure 6:**
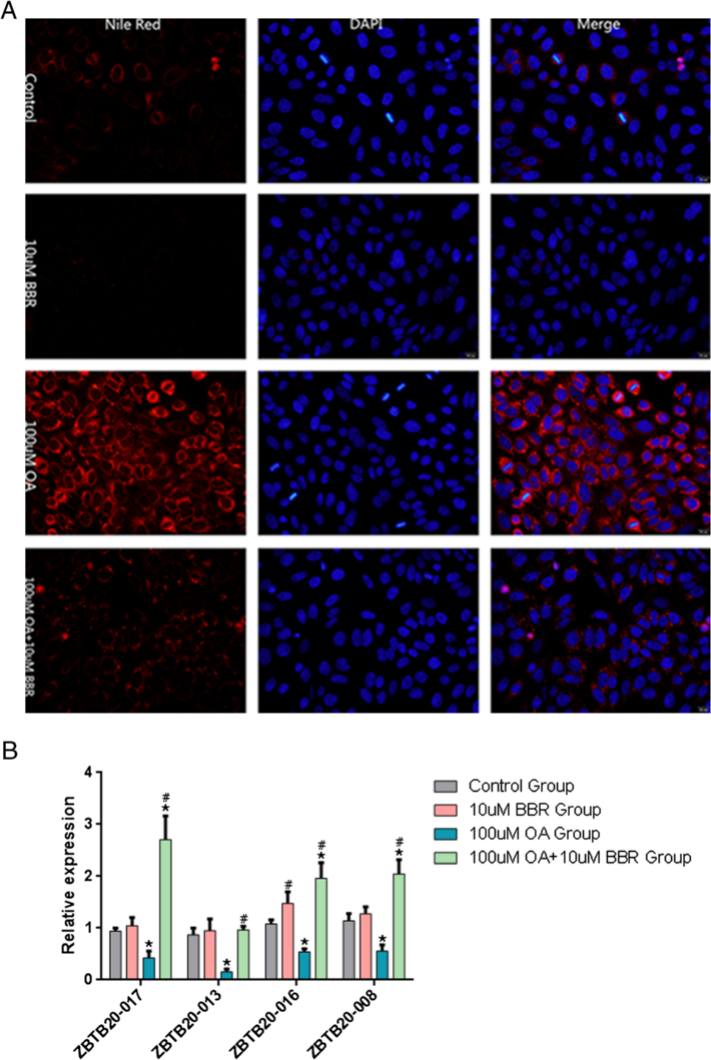
**The expression pattern of noncoding** ***ZBTB20*** **transcripts in human Huh7 cells. (A)** Huh7 cells were treated for 24 h with OA in the presence or absence of BBR (10 μM), and then stained with Nile Red. Photographs were taken by a fluorescence microscopy and shown at 400 × magnification. **(B)** Four human homologues of *MRAK052686* were measured by RT-qPCR in Huh7 cells treated with BBR, OA or BBR + OA as indicated (n = 3). Data are shown as mean ± SEM. *p < 0.05, vs control and ^#^p < 0.05, vs OA alone. Significance was assessed by one-way ANOVA followed by Tukey’s multiple comparison tests. OA, oleic acid; BBR, berberine.

By searching *MRAK052686* homolog in human genes, we found 6 homologous variants from ENSMBL database (Additional file 1: Table S1 and Additional file 2: Figure S1). Among 6 variants, four of them (i.e., *ZBTB20-008*, *-013*, *-016* and *-017*) were detected in human Huh7 hepatocytes (Figure 6). To further clarify whether the expression of these noncoding transcripts is also associated with hepatic steatosis in human, we treated Huh7 cells with oleic acid (OA) [26], which is regarded as an established steatotic cell model. As shown in Figure 6, cells exposed to 100 μM OA for 24 h resulted in an evident lipid accumulation, whereas BBR treatment significantly prevented the OA-induced steatosis (Figure 6A). In keeping with the findings from the HFD-fed animals, all the detectable variants of noncoding *ZBTB20* transcripts were significantly down-regulated in OA-treated hepatocytes, which were.completely reversed by BBR treatment (Figure 6B). Taken together, the findings not only indicate the connection of noncoding *ZBTB20* transcripts with hepatic steatosis, but also suggest that the protective effect of BBR against NAFLD is associated with the expression of these lncRNAs.

## Discussion

Multiple lines of evidences from both *in vivo* and *in vitro* studies have demonstrated a therapeutic potential of the botanic compound BBR in metabolic diseases, including obesity and diabetes [7,8,10-14]. In the present, we were able to confirm the effect of BBR in amelioration of NAFLD in HFD-induced obese rats as reported previously [12,14]. By analyzing gene expression profiles, we identified 5, 271 mRNAs and 3, 265 lncRNAs, which displayed differential expression patterns among the control, HFD and HFD + BBR treated animals. We further refined a large group of BBR-regulated genes, including 881 mRNAs and 538 lncRNAs whose expression was reversed between HFD and HFD + BBR groups, demonstrating a potent effect of BBR in globally altering hepatic gene expression. Moreover, by modularization of the BBR-regulated genes, we delineated several modules and identified numerous significant genes exhibiting a wide association with liver metabolism and NAFLD-related functions. The findings thus, for the first time, provide a genetic basis for understanding the pharmaceutical mechanism of BBR in protection against multiple metabolic diseases, including NAFLD.

Of interesting, not only were over 800 of BBR-regulated mRNAs identified in the current study, but also more than 500 aberrant lncRNAs in NAFLD were reversed by BBR treatment (Figure 2A). The number of altered lncRNAs was comparable with that of mRNAs. Although the precise roles of these lncRNAs in NAFLD are currently unknown, lncRNAs have emerged as important regulators of gene expression related to metabolic homeostasis or dysfunction. For instance, it was reported that near two hundreds of lncRNAs were regulated during the process of adipogenesis and many of them were bound at their promoters by key transcription factors such as PPARγ and CEBPα [40]. Furthermore, RNAi-mediated loss of function screens identified numerous lncRNAs that were required for proper adipogenesis [41]. There is a large body of evidences that lncRNAs can regulate gene expression both *in cis* and *in trans* by forming RNA-protein, DNA-RNA, protein-DNA and RNA-RNA interactions [41]. In the current study, we found that a total of 538 lncRNAs were regulated in the liver by BBR treatment in HFD-induced obese rats. Among these BBR-regulated lncRNAs, a set of lncRNAs was significantly associated with triglyceride trait (Additional file 8: Figure S6). In the analysis of co-expression sub-networks, we identified numerous lncRNAs were located in the salmon and dark golden modules, which are functionally linked with NAFLD-related genes (Figure 4 and Additional file 8: Figure S6). A significant example of these lncRNAs is *MRAK052686*. We found that *MRAK052686* expression associated with *Eif2ak2* and *Nrf2* (Figure 4), the two genes that are critically involved in NAFLD. By bioinformatics prediction, we found the interaction propensity and discriminative power of *Nrf2* and *Eif2ak2* interacting with *MRAK052686* are far stronger than that of the control genes *Actb* and *Gapdh* (Additional file 10: Figure S8). Indeed, a strong co-expression of *MRAK052686* and *Nrf2* was demonstrated by either microarray analysis or the experimental data from HFD-fed animals (Figure 5). This implies a potential role of *MRAK052686* in regulation of *Nrf2* expression.

*Nrf2* is a master transcription factor that regulates expression of many genes that are involved in antioxidant, metabolism and ER stress response [38,42]. In mammalian, *Nrf2* is regarded as a major cellular sensor of oxidative stress, which constitutes downstream the PERK (PKR-like ER-kinase) pathway during ER stress response [41]. Upon PERK activation, *Nrf2* is activated and binds to the antioxidant response elements in the regulatory regions of target genes, thereby regulating ER stress response [43,44]. Besides, *Eif2ak2*, another associated gene with *MRAK052686*, is actually a homologue of PERK. Moreover, *Gcs1* that was also co-expressed with *MRAK052686* functions in ER protein processing and regulates ER stress response. Indeed, ER stress has been well documented as a pivotal pathogenic event contributing to the process of hepatic steatosis [45,46]. The findings that *MRAK052686* associates with several key molecules of the ER stress response pathways suggest that *MRAK052686* may play important roles in NAFLD by affecting ER stress response, which warrants further investigations.

In the present study, another key finding is that most of the aberrantly expressed genes, including numerous lncRNAs, in HFD-induced hepatic steatosis were reversed by BBR treatment. In modularization of the sub-networks from salmon and dark golden modules, much more lncRNAs were co-expressed with enriched NAFLD-related mRNAs. This not only demonstrates a global effect of BBR on the alteration of gene profiles, but also suggests a population of lncRNAs as a whole target of the therapeutic action of BBR. Correspondingly, BBR exerts its effect to alleviate NAFLD through a systematic modulation of gene expression including lncRNAs in the liver, providing a potential novel therapeutic agent for the treatment of NAFLD.

## Conclusions

By analyzing hepatic gene expression profiles, we found that HFD-induced steatosis in rats resulted in a globe alteration in hepatic gene expression, which was significantly reversed by BBR treatment. Several modules of BBR-regulated genes, including abundant lncRNAs, were identified by bioinformatics analysis. Among these BBR-regulated genes, we found that the lncRNA *MRAK052686* and its associated gene *Nrf2* appear implicating in the pathogenesis of NAFLD. Thus, the study provides a new genetic insight into the pharmaceutical mechanism of BBR in protecting against NAFLD.

## Additional files

Additional file 1: Table S1. Sequence similarity of transcript variants of human homologous with rat *MRAK052686*. **Table S2.** The primers of genes for RT-qPCR. **Table S4.** Detailed function annotation of genes in seven modules. **Table S5.** Function annotation of mRNAs associated with *MRAK052686*. Additional file 2: Figure S1. Human chromosomal locations of *MRAK052686* and its homologs. The conserved regions between *MRAK052686* and its homologs were connected with dash lines. The thick and thin bars in gene models indicate protein-coding and non-protein coding regions, respectively. Additional file 3: Figure S2. A Venn diagram illustrates the number of genes that were differentially expressed in comparisons of HFD- or BBR-treated with ND group. The cyan arrows represent the comparison of HFD+BBR with ND group (HFD+BBR:ND) and grey arrows for the HFD:ND comparison. The upward and downward arrows indicate up-regulated and down-regulated expression, respectively. Additional file 4: Table S3. The list of BBR-regulated genes. Additional file 5: Figure S3. Hierarchical clustering tree of BBR-regulated genes. Each row represents the rat sample from ND (grey), HFD (purple) or HFD+BBR (cyan) group. The columns indicate BBR-regulated genes, which are divided into seven modules (the top color column from right to left: brown, blue, red, green, dark golden, light blue and salmon). Each cell of the heat-map reflects expression value above or below pool control level. The legend of expression value is shown on the upper left. Additional file 6: Figure S4. Relationship of gene modules with rat traits. Each row corresponds to a module eigengene, column to a trait. Each cell contains the corresponding correlation (p value, calculated by WGCNA package) and is colored by correlation according to the color legend on the right. CHOL, cholesterol; TG, triglyceride; HDL, high density lipoprotein; LDL, low density lipoprotein. The left color bars represent the modules consistent with Figure 3. Additional file 7: Figure S5. Gene significance of traits in salmon and red modules. **(A)** Gene significance of triglyceride in salmon modules. **(B)** Gene significance of LDL (low density lipoprotein) in red modules. The definition and calculation of gene significance and module correlation see Methods part. Circle and star points represent mRNAs and lncRNAs, respectively. The dash lines on the upper right are threshold sets for most significant genes labeled by larger markers and gene names. The linear fit curve with Pearson correlation (R) and significance (p value) of scatter points are shown. Additional file 8: Figure S6. Co-expression sub-network derived from enriched genes in salmon and red modules. Nodes of sub-network consist of enriched genes and their first neighboring genes. The threshold of co-expression network is 0.25 for weighted correlation. The red genes were identified by enrichment analysis showing in Figure 4. The potentially important lncRNAs are labeled in cyan color. Rectangles and circles represent lncRNAs and mRNAs, respectively. Additional file 9: Figure S7. Location and coding potential of *MRAK052686. MRAK052686* (blue) are transcribed from sense strand of protein-coding gene *Zbtb20* (green). The thick and thin bars of *Zbtb20* represent protein-coding and untranslated regions, respectively. The higher codon substitution frequency corresponds to stronger protein-coding potential. Additional file 10: Figure S8. The heat-map indicating an interaction of *MRAK052686* with *Nrf2* and Eif2ak2. The vertical index on the left of each heat-map is position of corresponding protein. IP and DP are abbreviations of interaction propensity and discriminative power, respectively. Higher IP and DP indicate more potential interaction.
