# Supplementary material for: Berberine ameliorates nonalcoholic fatty liver disease by a global modulation of hepatic mRNA and lncRNA expression profiles
Source: J Transl Med. 2015 Jan 27;13:24. doi: 10.1186/s12967-015-0383-6 (PMC4316752; doi:10.1186/s12967-015-0383-6)
Supplement: Additional file 1: Table S1. — Sequence similarity of transcript variants of human homologous with rat MRAK052686. Table S2. The primers of genes for RT-qPCR. Table S4. Detailed function annotation of genes in seven modules. Table S5. Function annotation of mRNAs associated with MRAK052686. [file 12967_2015_383_MOESM1_ESM.doc]

**Supplemental Tables**

**Supplemental Table 1. Sequence similarity of transcript variants of human homologous with rat *MRAK052686***

| Ensembl accession | Symbol | Location (chr: start-end, strand) | Query coverage | E value | Identity |
| --- | --- | --- | --- | --- | --- |
| ENST00000479879 | ZBTB20-017 | Chr3: 114339304-114900342, - | 17% | 3e-102 | 100% |
| ENST00000463890 | ZBTB20-013 | Chr3: 114380882-115100295, - | 10% | 4e-65 | 88% |
| ENST00000486152 | ZBTB20-016 | Chr3: 114387275-114500391, - | 7% | 8e-38 | 86% |
| ENST00000491500 | ZBTB20-015 | Chr3: 114388066-114900622, - | 7% | 3e-37 | 85% |
| ENST00000480832 | ZBTB20-014 | Chr3: 114388066-114861793, - | 7% | 3e-37 | 85% |
| ENST00000492665 | ZBTB20-008 | Chr3: 114389046-115100292, - | 4% | 2e-28 | 88% |

**Supplemental** Table 2. The primers of genes for RT-qPCR

| Gene | Strand | Primer (5’-3’) |
| --- | --- | --- |
| MRAK052686 | sense | Ccaccattttgatccacc |
|  | antisense | Gagcagcaacatcgcctc |
| Nfe2l2(Nrf2) | sense | gaataaagttgccgctcagaa |
|  | antisense | aaggtttcccatcctcatcac |
| ENST00000479879  (ZBTB20-017) | sense | gcacagactgcctgaagttaca |
|  | antisense | gactgatggtcaaagataggaataca |
| ENST00000463890  (ZBTB20-013) | sense | ggcagtcattaccactcacact |
|  | antisense | accttcagccttcagagtcc |
| ENST00000486152  (ZBTB20-016) | sense | caggagctgatgttgcctac |
|  | antisense | ttggaaagccaagtttctga |
| ENST00000491500  (ZBTB20-015) | sense | agcatttgagtttcagaggcc |
|  | antisense | ggtgcagtgatttcagtctctaaa |
| ENST00000480832  (ZBTB20-014) | sense | caatgcactccaattacaagaa |
|  | antisense | cattcttcgtaccctgtaggc |
| ENST00000492665  (ZBTB20-008) | sense | ctaagacagagctccttacaggg |
|  | antisense | ggtcacagactgatggtcaaag |
| Rat β-actin | sense | caggaaggaaggctggaaga |
|  | antisense | agagggaaatcgtgcgtgac |
| Human β-actin | sense | tgtgttggcgtacaggtctttg |
|  | antisense | gggaaatcgtgcgtgacattaag |

**Supplemental** Table 4. Detailed function annotation of genes in seven modules

| Module color | Count^*^ | Enriched biological processes of mRNAs (enriched count, p-value, Benjamini-adjusted p-value) |
| --- | --- | --- |
| Brown | 242, 61 | myofibril (10, 6.1E-6, 6.3E-4), extracellular region part (25, 1.4E-5, 7.5E-4), response to organic substance (29, 4.3E-5, 6.4E-2), cell adhesion (18, 1.7E-4, 0.12), ossification (8, 7.9E-4, 0.16), regulation of cell growth (9, 2.0E-3, 0.25), tube development (10, 6.4E-3, 0.42), regulation of adaptive immune response (5, 1.0E-2, 0.54) |
| Blue | 133, 27 | regulation of transcription, DNA-dependent (15, 4.2E-2, 0.96), regulation of cellular component size (6, 2.2E-2, 0.98), immune system development (7, 9.7E-3, 0.99) |
| Red | 105, 12 | extracellular matrix (11, 2.4E-6, 3.1E-4), melanosome (4, 9.6E-3, 0.23), Antigen processing and presentation (4, 2.0E-2, 0.30), **fatty acid metabolic process (6, 6.5E-3, 0.46)**, lymphocyte activation (8, 8.6E-5, 0.69), actin binding (6, 1.4E-2, 0.96) |
| Green | 62, 44 | G-protein coupled receptor protein signaling pathway (19, 2.2E-5, 7.4E-3), pheromone receptor activity (4, 3.4E-3, 0.28) |
| Dark golden | 139, 175 | **fat-soluble vitamin metabolic process (3, 3.3E-2, 0.97)**, **response to vitamin (5, 1.2E-2, 1.00)**, determination of left/right symmetry (3, 3.1E-2, 1.00) |
| Light blue | 81, 103 | olfactory receptor activity (12, 1.2E-2, 0.87), organelle localization (3, 4.4E-2, 0.88) |
| Salmon | 119, 116 | amine receptor activity (6, 4.7E-6, 8.4E-4), G-protein coupled receptor protein signaling pathway (29, 1.4E-5, 1.1E-2), positive regulation of catalytic activity (9, 5.6E-3, 0.14), enzyme activator activity (5, 3.0E-2, 0.84), **endoplasmic reticulum (8, 2.2E-1, 0.90)** |

Note, ^*^ The first and second numbers represent count of mRNAs and lncRNAs, respectively.

***Supplemental Table 5. Function annotation of mRNAs associated with MRAK052686***

| Gene | Correlation | Gene ontology |
| --- | --- | --- |
| Eif2ak2 | 0.59 | Protein processing in endoplasmic reticulum |
| Adh1 | 0.59 | Fatty acid degradation, Drug metabolism - cytochrome P450 |
| Nfe2l2 | 0.91 | Protein processing in endoplasmic reticulum |
| Ap2a2 | 0.74 | Lipid binding, vesicle-mediated transport |
| Gcs1 | 0.47 | Protein processing in endoplasmic reticulum |
| Mbl2 | 0.37 | Complement activation, lectin pathway |
| Fabp7 | 0.36 | PPAR signaling pathway, fatty acid binding |
| Apba1 | -0.02 | intracellular protein transport |
