# Supplementary figures and images for: Berberine ameliorates nonalcoholic fatty liver disease by a global modulation of hepatic mRNA and lncRNA expression profiles
Source: J Transl Med. 2015 Jan 27;13:24. doi: 10.1186/s12967-015-0383-6 (PMC4316752; doi:10.1186/s12967-015-0383-6)

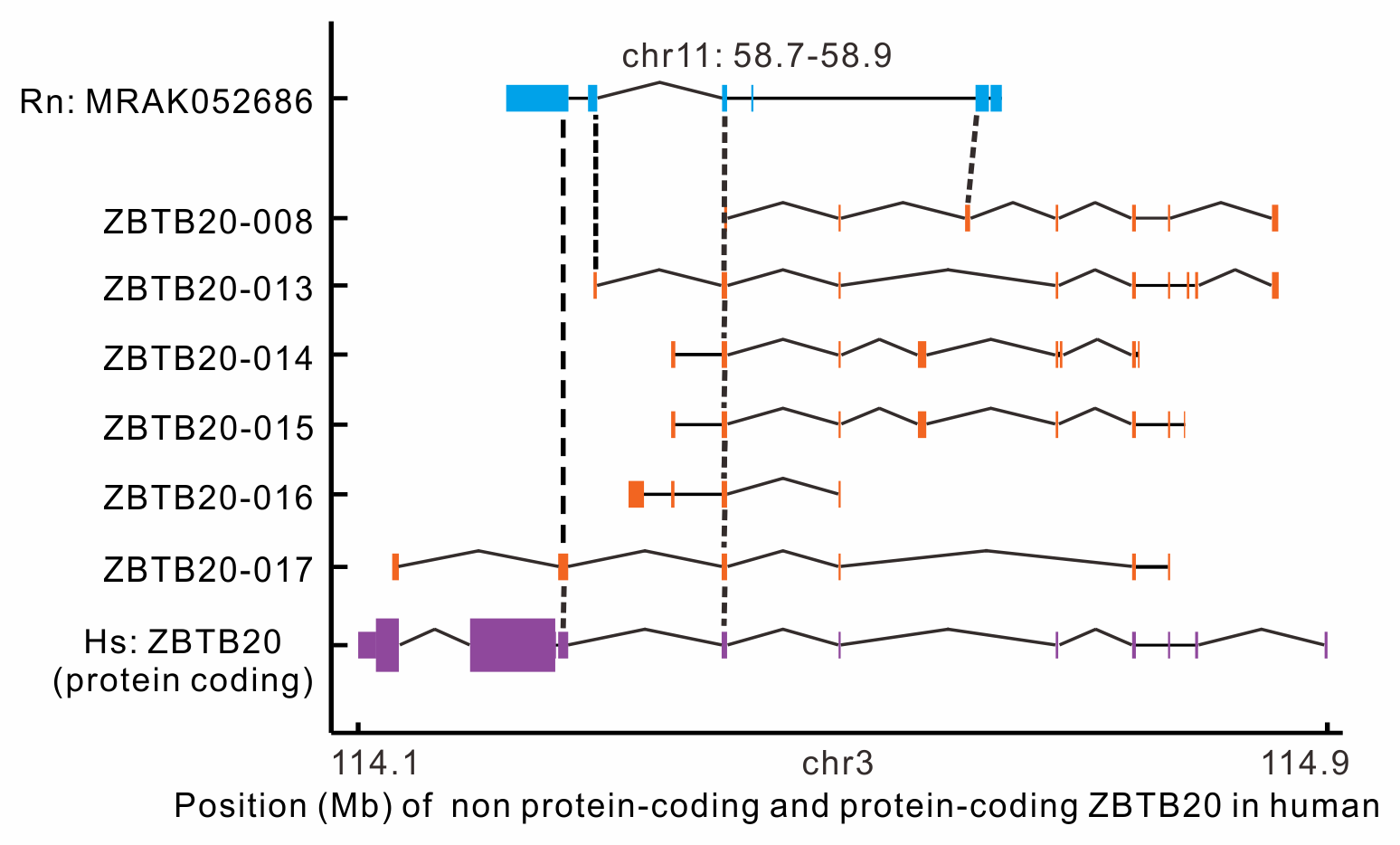

Supplement: Additional file 2: Figure S1. — Human chromosomal locations of MRAK052686 and its homologs. The conserved regions between MRAK052686 and its homologs were connected with dash lines. The thick and thin bars in gene models indicate protein-coding and non-protein coding regions, respectively. [file 12967_2015_383_MOESM2_ESM.tiff]

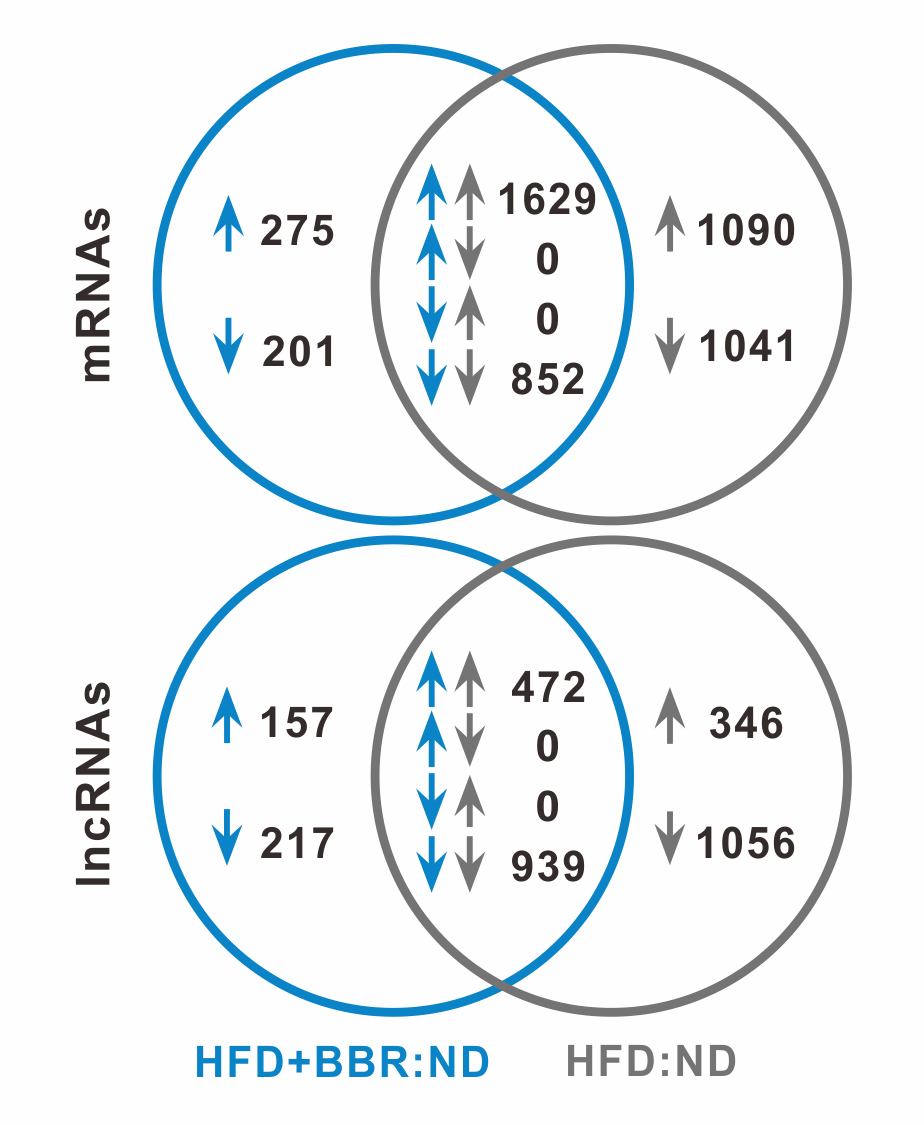

Supplement: Additional file 3: Figure S2. — A Venn diagram illustrates the number of genes that were differentially expressed in comparisons of HFD- or BBR-treated with ND group. The cyan arrows represent the comparison of HFD+BBR with ND group (HFD+BBR:ND) and grey arrows for the HFD:ND comparison. The upward and downward arrows indicate up-regulated and down-regulated expression, respectively. [file 12967_2015_383_MOESM3_ESM.tiff]

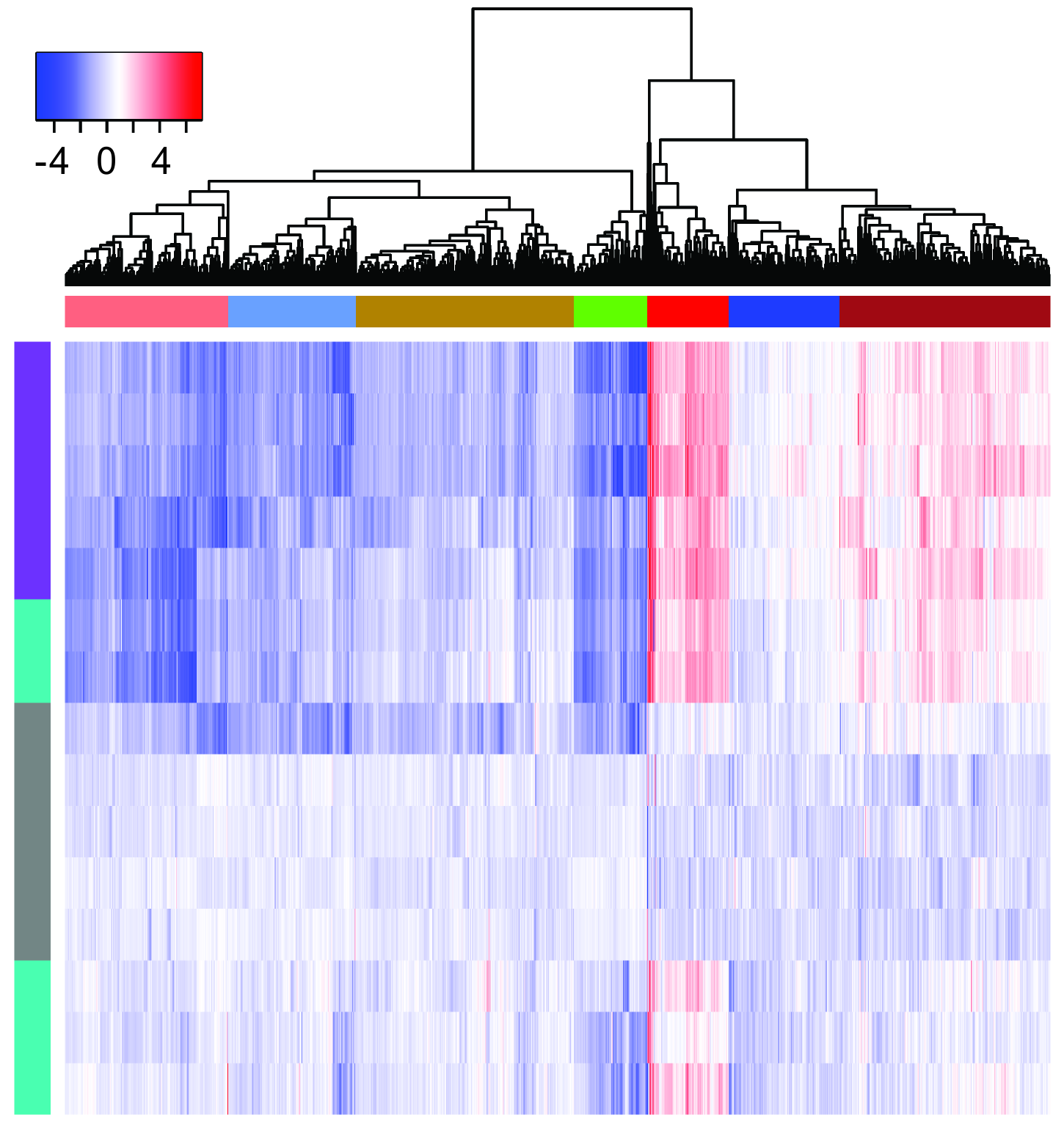

Supplement: Additional file 5: Figure S3. — Hierarchical clustering tree of BBR-regulated genes. Each row represents the rat sample from ND (grey), HFD (purple) or HFD+BBR (cyan) group. The columns indicate BBR-regulated genes, which are divided into seven modules (the top color column from right to left: brown, blue, red, green, dark golden, light blue and salmon). Each cell of the heat-map reflects expression value above or below pool control level. The legend of expression value is shown on the upper left. [file 12967_2015_383_MOESM5_ESM.tiff]

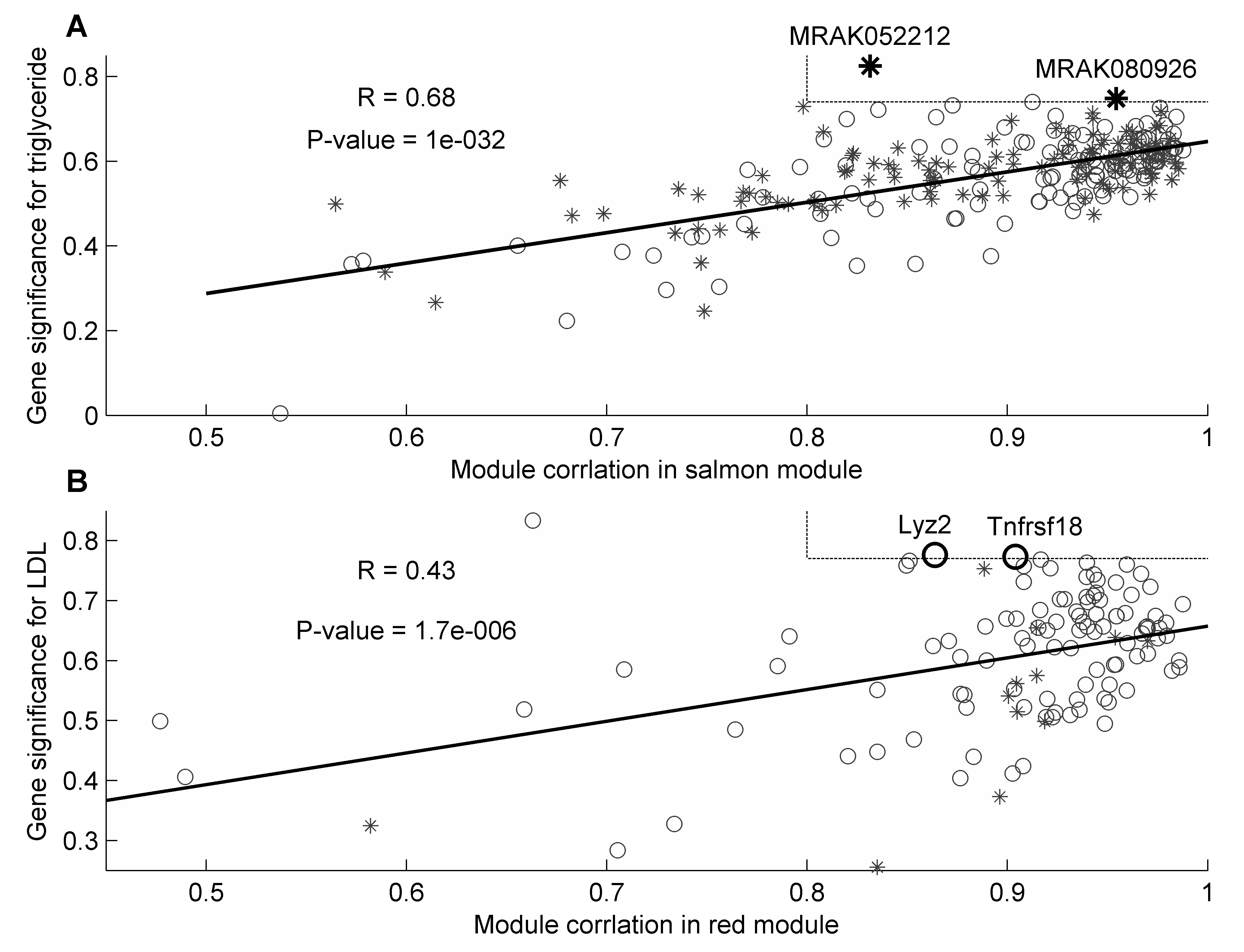

Supplement: Additional file 7: Figure S5. — Gene significance of traits in salmon and red modules. (A) Gene significance of triglyceride in salmon modules. (B) Gene significance of LDL (low density lipoprotein) in red modules. The definition and calculation of gene significance and module correlation see Methods part. Circle and star points represent mRNAs and lncRNAs, respectively. The dash lines on the upper right are threshold sets for most significant genes labeled by larger markers and gene names. The linear fit curve with Pearson correlation (R) and significance (p value) of scatter points are shown. [file 12967_2015_383_MOESM7_ESM.tiff]

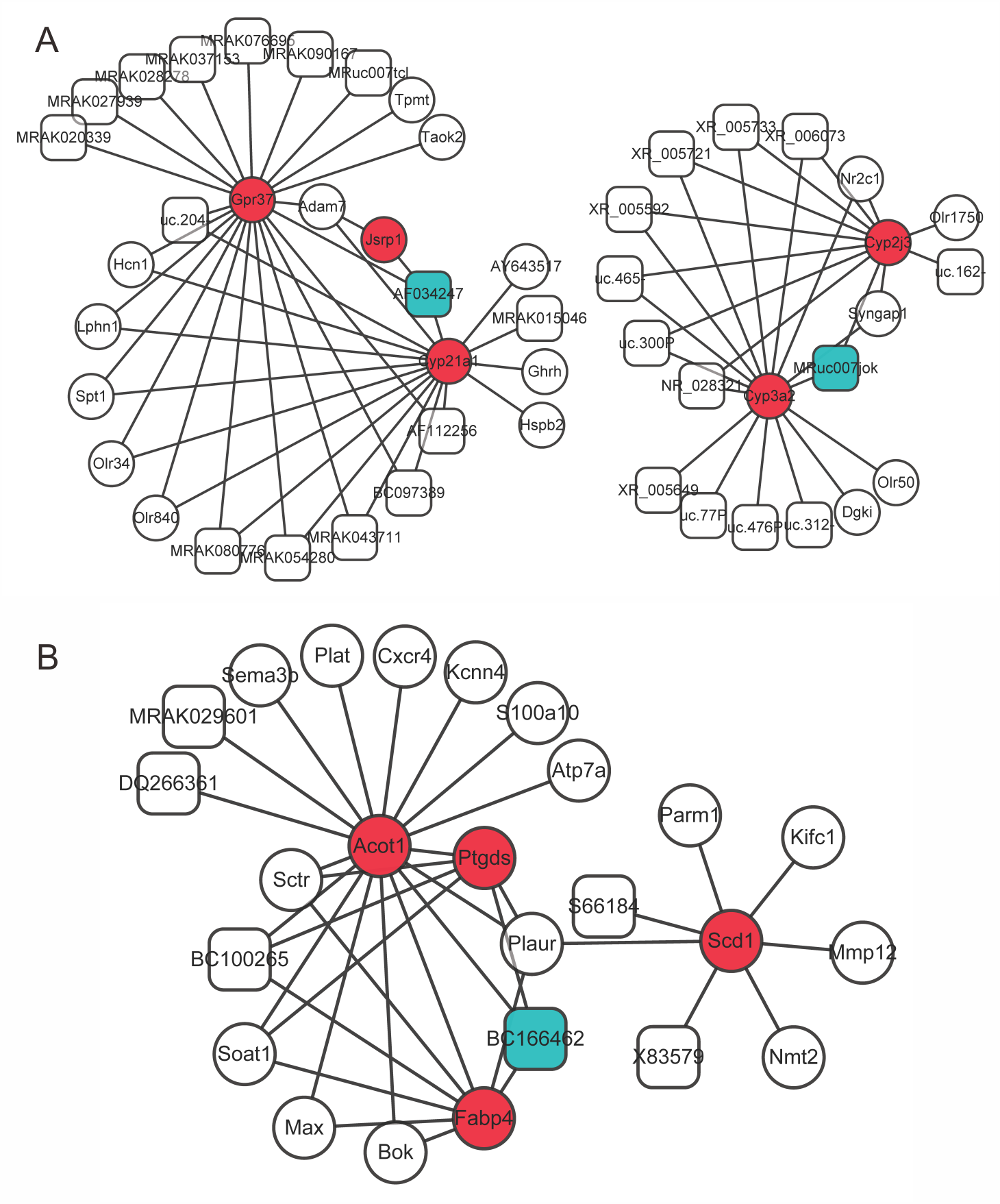

Supplement: Additional file 8: Figure S6. — Co-expression sub-network derived from enriched genes in salmon and red modules. Nodes of sub-network consist of enriched genes and their first neighboring genes. The threshold of co-expression network is 0.25 for weighted correlation. The red genes were identified by enrichment analysis showing in Figure 4. The potentially important lncRNAs are labeled in cyan color. Rectangles and circles represent lncRNAs and mRNAs, respectively. [file 12967_2015_383_MOESM8_ESM.tiff]

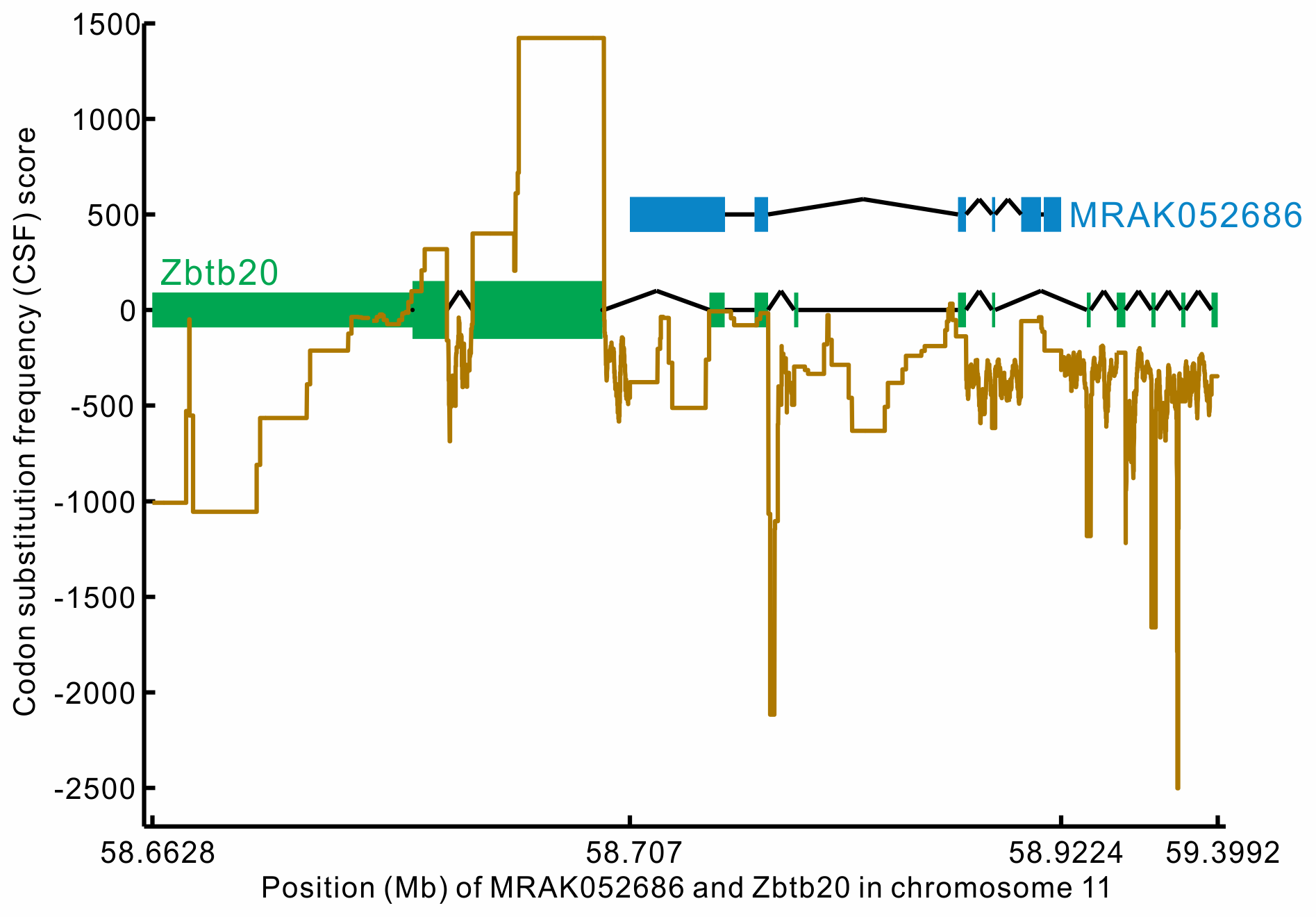

Supplement: Additional file 9: Figure S7. — Location and coding potential of MRAK052686. MRAK052686 (blue) are transcribed from sense strand of protein-coding gene Zbtb20 (green). The thick and thin bars of Zbtb20 represent protein-coding and untranslated regions, respectively. The higher codon substitution frequency corresponds to stronger protein-coding potential. [file 12967_2015_383_MOESM9_ESM.tiff]

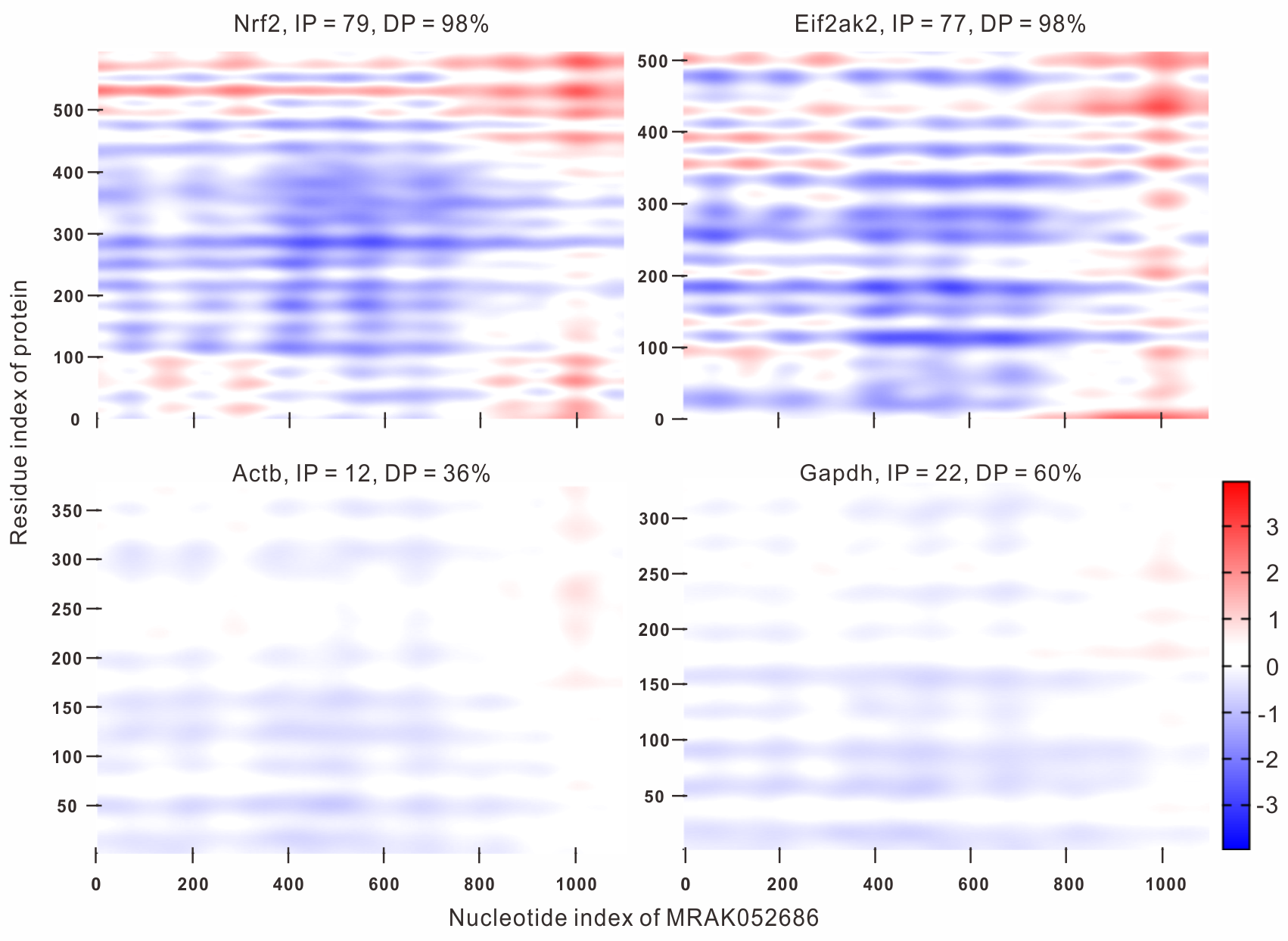

Supplement: Additional file 10: Figure S8. — The heat-map indicating an interaction of MRAK052686 with Nrf2 and Eif2ak2. The vertical index on the left of each heat-map is position of corresponding protein. IP and DP are abbreviations of interaction propensity and discriminative power, respectively. Higher IP and DP indicate more potential interaction. [file 12967_2015_383_MOESM10_ESM.tiff]
